# 4-Acetoxydolastane Diterpene from the Brazilian Brown Alga *Canistrocarpus cervicornis* as Antileishmanial Agent

**DOI:** 10.3390/md9112369

**Published:** 2011-11-11

**Authors:** Adriana Oliveira dos Santos, Elizandra Aparecida Britta, Everson Miguel Bianco, Tania Ueda-Nakamura, Benedito Prado Dias Filho, Renato Crespo Pereira, Celso Vataru Nakamura

**Affiliations:** 1 Postgraduate Program in Microbiology, State University of Londrina, Highway Celso Garcia Cid, PR 445, Km 380, CEP 86051-990, Londrina, Parana, Brazil; E-Mails: oi_dri@hotmail.com (A.O.d.S.); bdpfilho@uem.br (B.P.D.F.); 2 Postgraduate Program in Pharmaceutical Sciences, Laboratory of Technological Innovation in the Development of Drugs and Cosmetics, State University of Maringa, Colombo Avenue 5790, CEP 87020-900, Maringa, Parana, Brazil; E-Mails: elizandrabritta@hotmail.com (E.A.B); tunakamura@uem.br (T.U.-N.); 3 Postgraduate Program in Chemistry, Department of Fundamental Chemistry, Federal University of Pernambuco, CEP 50670-901, Recife, Pernambuco, Brazil; E-Mail: ebianco@chemist.com; 4 Department of Marine Biology, Federal Fluminense University, PO Box 100644, CEP 24001-970, Niteroi, Rio de Janeiro, Brazil; E-Mail: egbrecp@vm.uff.br

**Keywords:** antileishmanial activity, *Leishmania amazonensis*, *Canistrocarpus cervicornis*

## Abstract

Natural marine products have shown an interesting array of diverse and novel chemical structures with potent biological activities. Our study reports the antiproliferative assays of crude extracts, fraction and pure compound (4*R*,9*S*,14*S*)-4α-acetoxy-9β,14α-dihydroxydolast-1(15),7-diene (**1**) obtained from brown alga *Canistrocarpus cervicornis* showing the antileishmanial activity. We showed that **1** had a dose-dependent activity during 72 h of treatment, exhibiting IC_50_ of 2.0 μg/mL, 12.0 μg/mL, and 4.0 μg/mL for promastigote, axenic amastigote and intracellular amastigote forms of *Leishmania amazonensis*, respectively. A cytotoxicity assay showed that the action of the isolated compound **1** was 93.0 times less toxic to the macrophage than to the protozoan. Additionally, compound **1** induced ultrastructural changes, including extensive mitochondrial damage; decrease in Rh123 fluorescence, suggesting interference with the mitochondrial membrane potential; and lipid peroxidation in parasite cells. The use of **1** from *C. cervicornis* against *L. amazonensis* parasites might be of great interest as a future alternative to the development of new antileishmanial drugs.

## 1. Introduction

Human leishmaniasis is an endemic parasitic disease which represents a major health problem in the tropical and subtropical regions of the world. The prevalence is 12 million people and the overall population at risk is 350 million people [1].

Leishmaniasis is caused by a species of the *Leishmania*, a flagellated protozoan from the *Trypanosomatidae* family. The disease is transmitted by the bite of a sandfly which belongs to *Lutzomia genus*. Parasites of the *Leishmania* sp. have a heteroxenic life cycle that includes an intracellular amastigote form within the mononuclear phagocytes in mammal hosts and an extracelular promastigote form in vector insects [2,3].

Leishmaniasis is classified in four distinct clinical forms, visceral (VL), cutaneous (CL), difuse cutaneous (DCL) and mucocutaneous leishmaniasis (MCL) [4]. The clinical manifestations depend on the parasite species and the susceptibility of the host. The therapy is very complicated because the drugs for the treatment have many limitations and *Leishmania* species have already presented resistence to the drugs [2]. Pentavalent antimonials have been used for leishmaniasis treatment for more than 50 years. However, they cause serious side effects, such as cardiac and renal toxicity, as well as continuing to require long-term treatment. Other alternative drugs to antimonials in unresponsive cases are: Pentamidine and amphotericin B. Unfortunately, these drugs also cause toxic effects [5,6].

Bioactive natural products have been isolated from marine organisms and their pharmacological properties analyzed [7]. For example, brown algae produce a range of these compounds that have a wide variety of ecological functions such as defense against herbivores [8], fouling [9,10] and pathogenic microbes [11]. They also display a wide spectrum of pharmacological properties, such as antiviral [12], antiprotozoa [13,14], antibacterial [15], antioxidant [16] and anticoagulant [17].

A variety of different dolastane and *seco*-dolastanes diterpenes have been isolated from the brown marine macroalga *Canistrocarpus cervicornis* (formerly *Dictyota cervicornis*) [10,18–21]. In this study the leishmanicidal activity of crude extracts, fraction, and a 4-acetoxy-dolastane diterpene (**1**) obtained from *C. cervicornis* were first measured in laboratory assays against the *Leishmania amazonensis*.

## 2. Results and Discussion

Marine brown algae of the family *Dictyotaceae* are rich sources of monocyclic, bicyclic, and tryciclic diterpenes as major secondary metabolites [18,22–26]. Interestingly, this seaweed has a wide distribution along the Brazilian coast [25]. This traditional medicine still plays an important role in primary health care [27]. Plant-derived drugs remain an important resource, especially in developing countries, in combating diseases [6]. This is particularly true for marine natural products, which show an interesting array of diverse and novel chemical structures with potent biological activities [28].

**Figure 1:**
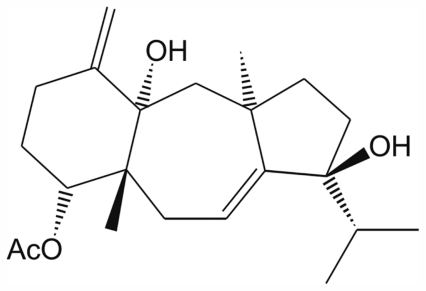
Chemical structure of compound **1** (4*R*,9*S*,14*S*)-4α-acetoxy-9β,14α-dihydro xydolast-1(15),7-diene, isolated from *Canistrocarpus cervicornis*.

**Figure 2:**
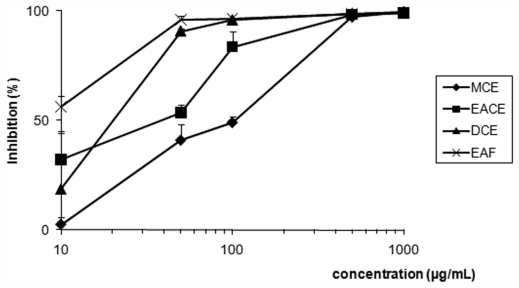
Effect of crude extracts (EACE, MCE and DCE), and fraction (EAF) from brown alga *Canistrocarpus cervicornis* against promastigote forms of *Leishmania amazonensis*. Each bar represents one standard deviation.

**Figure 3:**
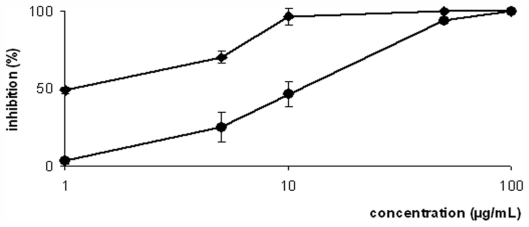
Effect of compound **1** from brown alga *Canistrocarpus cervicornis* against promastigote (◆), and axenic amastigote (●) forms of *Leishmania amazonensis*. Each bar represents one standard deviation.

Our study reports the antiproliferative assays of crude extracts (EACE, MCE and DCE), fraction (EAF) and pure compound (**1**) (4*R*,9*S*,14*S*)-4α-acetoxy-9β,14α-dihydroxydolast-1(15),7-diene (Figure 1) obtained from brown alga *C. cervicornis* showing the dose-dependent effect against promastigote forms of *L. amazonensis* (Figures 2 and 3). In Table 1, we have demonstrated the concentrations values of crude extracts, fraction, and **1** that inhibited 50% of this parasite (IC_50_ values) after 72 h of incubation. Thus, the IC_50_ of crude extracts EACE, MCE and DCE were 50.0 μg/mL, 100.0 μg/mL and 20.0 μg/mL, respectively. The IC_50_ of fraction (EAF) and **1** were 8.0 μg/mL and 2.0 μg/mL, respectively. The student’s *t* test (*p* < 0.05) indicated significant differences between crude extracts, fraction and isolated compounds compared to the control group. In addition, amphotericin B showed IC_50_ of 0.06 μg/mL against promastigote forms after 72 h of treatment.

The cytotoxicity on macrophage strain J774G8 of crude extracts, fraction, and isolated compound were also evaluated (Table 1). When macrophages were treated with crude extracts EACE, MCE, and DCE the 50% cytotoxic concentration (CC_50_) were 50.0 μg/mL, 51.0 μg/mL, and 46.0 μg/mL, respectively. The CC_50_ of fraction (EAF) was 47.0 μg/mL and CC_50_ of **1** was 186.0 μg/mL. The cytotoxicity to the macrophage and the activity against the protozoan were compared by using the selectivity index (SI). The better results of SI for promastigote forms were obtained with fraction EAF (SI = 5.88) and isolated compound **1** (SI = 93.0). So, EAF and **1** were respectively 5.88 and 93.0 times less toxic to the macrophage than to the protozoan.

**Figure 4:**
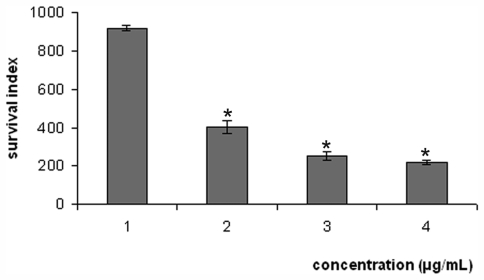
Survival index of *Leishmania amazonensis* within peritoneal macrophage cells treated with compound **1**. (1) Untreated control; (2) Treated with **1** at 5.0 μg/mL; (3) Treated with **1** at 15.0 μg/mL; (4) Treated with **1** at 30.0 μg/mL. Each bar represents the mean ± standard error of at least three independent experiments, which were performed in duplicate. * Significant difference of each group from the control (*p* < 0.05).

The pure compound **1** was more active than crude extracts and fraction. Thus, further experiments were carried out using only this compound. Isolated compound **1** had the activity investigated against axenic amastigote forms of *L. amazonensis* (Figure 3). The IC_50_ obtained value was 12.0 μg/mL. The effect of the **1** on intracellular amastigotes was observed during 24 h of incubation (Figure 4). Treatment of intracellular amastigotes with 5.0, 15.0, and 30.0 μg/mL with **1** resulted in decreases in the survival index of 56.0, 72.0, and 76.0%, respectively, compared to the control. Additionally, the IC_50_ was 4.0 μg/mL. These results were significant at *p* ≤ 0.05 as compared to the control group, by the student’s *t* test. In this regard, the result of this preliminary study is very encouraging because it is the amastigote forms of *Leishmania* that persist in the human host and directly cause all of the clinical manifestations of leishmaniasis [29]. Amphotericin B showed IC_50_ of 0.25 and 0.35 μg/mL against axenic amastigote and intracellular amastigote forms, respectively.

**Figure 5:**
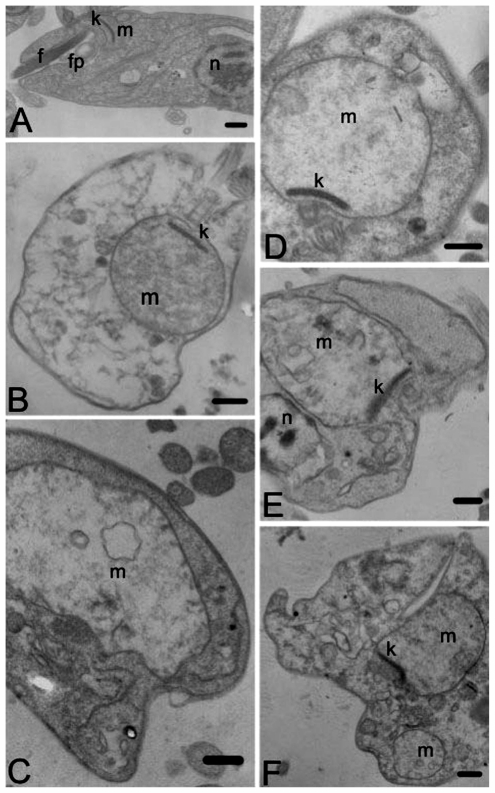
Ultrastructural effect of compound **1** after incubation for 72 h at 25 °C on promastigotes forms of *Leishmania amazonensis* observed by Transmission Electron Microscopy. (**A**) Promastigote Control; (**B** and **C**) Promastigote forms treated with 2 μg/mL of **1**; (**D** to **F**) Promastigote forms treated with 10 μg/mL of **1**. The treatment led to notable swollen mitochondrial (black stars). n, nucleus; f, flagellum; fp, flagellar pocket; k, kinetoplast; m, mitochondrion. Bars = 1 μm.

To find cellular targets in leishmania cells treated with **1**, TEM and flow cytometry techniques were employed. We verified that **1** produced ultrastructural alterations in *L. amazonensis.* Figure 5 shows transmission electron microscopy photomicrographs of promastigotes treated with the isolated compound **1**. Figure 5A shows untreated cells with a terminal flagellum, nucleus, and the single mitochondrion with a branched structure, characteristic of this group of organisms, containing a disk-shaped aggregate of DNA called a kinetoplast. In *L. amazonensis* treated with **1**, we highlight that the main alteration which occurred in the mitochondria of the parasite, was seen as intense mitochondrial swelling, as seen in Figure 5B–F.

**Figure 6:**
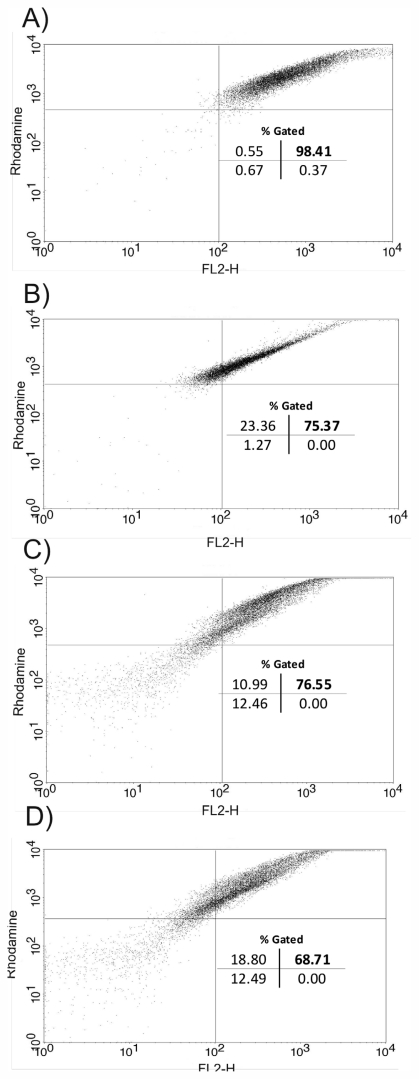
Flow cytometry analysis of Rh123-labeled axenic amastigotes of *Leishmania amazonensis*. Compound **1** collapsed the ΔΨm, leading to parasite death. (**A**) Untreated cells; (**B**) CCCP 200 μM; (**C**) Amastigotes treated with 50 μg/mL; (**D**) Amastigotes treated with 100 μg/mL. The bold numbers represent the percentage of the collapsed ΔΨm cells in the upper right quadrant.

In accordance with these findings, tests using specific markers for mitochondria were performed. Data obtained from a flow cytometry by using Rh123 presented in Figure 6 showed a marked decrease in the percentage population of upper right gate (76.55% and 68.71%). This indicates depolarization of the mitochondrial membrane potential in the cells following treatment with **1** at 50 μg/mL and 100 μg/mL, respectively (Figure 6). Similarly, a decrease in membrane potentials was also observed following treatment with the standard drug Carbonyl Cyanide *m*-chlorophenylhydrazone (CCCP) (75.37%) at 200 μM for 3 h at 32 °C. In contrast, untreated cells maintained the membrane potential (98.41%) (Figure 6, upper right quadrant). The student’s *t* test (*p* < 0.05) indicated significant differences between cells treated with copaiba oil compared to the negative control group.

Mitochondrion found in trypanosomatid parasites had distinct structural and functional characteristics of mammalian cells making this organelle an exceptionally attractive chemotherapeutic target. Likewise, investigations have shown that mitochondria can be targeted by different drugs for *Leishmania* sp. [13,14,30–32]. Additionally, several studies have reported that mitochondrial alterations may lead to programmed cell death by apoptosis [33–36].

**Figure 7:**
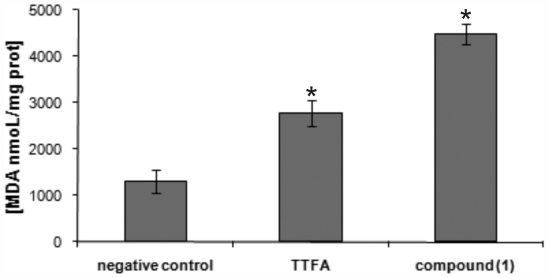
Effect of compound **1** on lipid peroxidation (production of malondialdehyde (MDA)) of amastigote forms of *Leishmania amazonensis*. Each bar represents the mean ± standard error of at least three independent experiments, which were performed in duplicate. * Significant difference of each group from the control (*p* < 0.05).

In order to evaluate the effects of the compound **1**, the lipid peroxidation of cell membrane was determined. It was assessed by measuring TBARS in leishmanial cells after treatment with **1** compared to control or untreated cells (Figure 7). In addition, thenoyltrifluoroacetone (TTFA), a conventional mitochondrial complex II inhibitor, was used as positive control. Compound **1** treatment at 100 μg/mL displayed an increase in lipid peroxides after 6 h of drug treatment, with a 3.4-fold lipoperoxidation compared to the untreated control cells while the increase in lipid peroxidation obtained with the TTFA was 2.1-fold compared to the control. Previous studies have demonstrated that loss of the mitochondrial membrane potentially induces formation of reactive oxygen species (ROS) inside cells, which induces lipid peroxidation. Moreover, the cellular ROS generation in turn leads to the damage of the oxidative citoplasmatic membrane, and DNA lesions [33,34,37,38].

## 3. Experimental Section

### 3.1. Algal Collection

Specimens of the brown macroalga *C. cervicornis* (Dictyotaceae, Phaeophyta) were collected by free diving off the coast of Paraíso Beach, Pernambuco State, Brazil (08°21′S, 34°57′W), in October 2009, at depths between 1 and 2 m. The algal material was cleaned manually from epiphytic organisms and air-dried immediately after collection. Voucher specimens were deposited in the Herbarium of Universidade Federal de Pernambuco, Recife, Brazil (62948).

### 3.2. Chemical Extraction

The air-dried algal material (300.0 g of *C. cervicornis*) was extracted in dichloromethane (CH_2_Cl_2_) at room temperature for 30 days and the solvent was evaporated *in vacuo* by rota-evaporation, yielding 14.6 g of dry dichloromethane crude extract (DCE). The residue was further extracted in ethyl acetate (EtOAc) using similar procedures, yielding 0.9 g of dry ethyl acetate crude extract (EACE), and finally extracted in methanol (MeOH), yielding 0.3 g of dry methanol crude extract (MCE).

### 3.3. Fractionation and Compound Isolation

A part of DCE (5 g) was subjected to silica gel 70–230 mesh column chromatography (3.5 × 25 cm), eluted with *n*-hexane, CH_2_Cl_2_, EtOAc, Me_2_CO, and MeOH in sequence, to give 45 fractions of 20 mL each one (F1–F45). The fractions F12–F25 eluted with CH_2_Cl_2_ showed similar when analyzed by TLC and it were combined to produce a dark brown-green residue (1.2 g), which was subjected to new silica gel 70–230 mesh column chromatography (2 × 25 cm) eluted with *n*-hexane/EtOAc (3:1) increasing the polarity until 100% AcOEt, to give 48 fractions (F1–F48, 10 mL each one). The fractions F14–F18 were analyzed by TLC and showed intense browns spots (Rf = 0.47, mobile phase *n*-hexane/EtOAc 3:1), then it was combined (200 mg). Following this, fractions were subjected to new silica gel 70–230 mesh column chromatography (1.0 × 20 cm), eluted with *n*-hexane/EtOAc (4:1), to give 20 fractions (F1–F20).

The Fraction F8 afforded the pure compound (4*R*,9*S*,14*S*)-4α-acetoxy-9β,14α-dihydroxydolast-1(15), 7-diene (**1**) (143.0 mg, yellow gum). The fractions F26–F38 eluted with EtOAc from DCE showed similar results when analyzed by TLC, and then they were combined to produce a brownish residue (1.3 g) which was named EAF (ethyl acetate fraction).

All the crude extracts formed (EACE, DCE, and MCE), fraction (EAF) and pure compound isolated **1** were monitored by TLC using silica gel GF_254_ (Merck) as stationary phase and *n*-hexane/EtOAc 3:1 and 4:1 as mobile phase. The chromatoplates used were revealed through spraying it with a solution of ceric sulphate and sulfuric acid acquired by 2.1 g of Ce_2_(SO_4_)_3_.4H_2_O; 21 mL of H_2_SO_4_ and 300 mL of H_2_O, followed by heating at 100 °C for 3 min.

### 3.4. Spectroscopic Data

The physical and spectroscopic properties of the diterpene (4*R*,9*S*,14*S*)-4α-acetoxy-9β,14α-dihydro xydolast-1(15),7-diene (143.0 mg, yellowish gum) were found to be identical with the previously reported data [39].

### 3.5. Parasites

*Leishmania* promastigotes were grown in Warren’s medium (brain-heart infusion plus hemin and folic acid) pH 7.2, supplemented with 10% heat-inactivated fetal bovine serum (FBS; Gibco Invitrogen Corporation, New York, USA), at 24 °C in a tissue flack. The strain used was *Leishmania amazonensis* (MHOM/BR/Josefa) originally isolated from a human case of difuse cutaneous leishmaniasis.

*Leishmania* axenic amastigotes were obtained by *in vitro* transformation of infective promastigotes [40]. These forms were maintained in Schneider’s medium (Sigma, St. Louis, MO, USA) pH 4.6, supplemented with 20% heat-inactivated FBS, at 32 °C in a tissue flask.

### 3.6. Macrophage J774G8 Cells

Murine macrophage J774G8 cells were maintained in tissue flasks composed of RPMI 1640 medium (Gibco Invitrogen Corporation, New York, USA), sodium bicarbonate, l-glutamine, and supplemented with 10% heat-inactivated FBS in a 5% CO_2_–air-mixture.

### 3.7. Antileishmania Activity *in Vitro* against Promastigotes and Axenic Amastigotes

The growth inhibition test that was performed on promastigotes forms of *L. amazonensis* from a 48 h logarithmic phase culture was suspended to yield 1 × 10^6^ parasites/mL. It was then cultivated in 24-well culture plates at 25 °C in Warren’s medium, supplemented with 10% FBS in the presence or absence of increasing concentration of **1** for 72 h. In a same way, axenic amastigotes forms of *L. amazonensis* from 72-h-old logarithmic-phase culture were suspended to yield 1 × 10^6^ parasites/mL, and then were cultivated in 12-well culture plates at 32 °C in Schneider’s medium. Next they were supplemented with 20% FBS in the presence or absence of increasing concentration of compound **1** for 72 h. Amphotericin was used as a positive control. DMSO was used for the solubilized drugs, but the final DMSO concentration did not exceed 0.5%, which did not show deleterious effects on the parasites. The leishmanicidal activity was determined by direct counting of the cells in a Neubauer chamber and the 50% inhibition concentration (IC_50_) was obtained graphically by plotting concentration *versus* percentage of growth inhibition.

### 3.8. Activity against Intracellular Amastigotes

For assay of the effects of the compound on intracellular amastigotes, peritoneal macrophages of BALB/C, mice were used. Peritoneal macrophages (5 × 10^5^ cells/mL) were plated on coverslips (diameter 13 mm) in 24 well plate in RPMI 1640 medium supplemented with 10% inactive FBS, and incubated for 24 h at 37 °C in a 5% CO_2_ atmosphere for adherence, after the peritoneal macrophages were infected with promastigotes of *L. amazonensis* in multiples of 10 parasites per host cell and incubated at 37 °C in a 5% CO_2_ atmosphere. After 6 h of infection, infected macrophages were treated with compound in concentrations 10, 20 and 30 μg/mL and incubated 24 h again. Afterwards the monolayer’s were washed with PBS, fixed with methanol, and stained with Giemsa. The percentage of infected macrophages and the mean numbers of amastigotes/infected macrophage were determined by counting at least 200 macrophages in duplicate cultures, and results were expressed as shown in the survival index. The survival index was obtained by multiplying the percentage of macrophages cells with parasites by the mean number of internalized parasites per cell.

### 3.9. Cytotoxicity Assay

The cytotoxicity assay was performed in 96-well plates. A suspension of 5 × 10^5^ J774G8 cells in RPMI 1640 medium supplemented with 10% FBS was added to each well in 96-well microtiter plates. The plates were incubated in a 5% CO_2_–air mixture at 37 °C to obtain confluent growth of the cells. After 24 h, the medium was removed and the cells were treated with several concentrations of compounds and the plates were incubated for 48 h. Control cells without compound were included. The adhered macrophages were fixed with 50 μL/well of 10% trichloroacetic acid at 4 °C for 1 h; after that, the well plates were washed with water, and attained with 50 μL/well of sulforhodamine B (0.4% w/v) in 1% acetic acid solution; the microplate was then maintained at 4 °C for 30 min. Next, the microplate was washed five times with 1% acetic acid to remove the sulforhodamine B, then 150 μL/well of 10 mM unbuffered Tris-base solution (Sigma) was added. The absorbance of each individual well was read at 530 nm. Dose-response curves were plotted (values expressed as percentage of control optical density) and the 50% cytotoxicity concentration (CC_50_) was determined by logarithm regression analysis of the data obtained.

### 3.10. Transmission Electron Microscopy

Ultrastructural analysis was performed on promastigote forms of *L. amazonensis* treated with 2.0 and 10.0 μg/mL of the compound **1**, after 48 h for treatment, the parasites were washed in 0.1 M phosphate-buffered saline and fixed in 2.5% glutaraldehyde in 0.1 M sodium cacodylate buffer at 4 °C. The cells were post fixed in a solution containing 1% osmium tetroxide, 0.8% potassium ferrocyanide, and 5 mM calcium chloride in 0.1 M cacodylate buffer. Next they were washed in the same buffer, dehydrated in increasing concentrations of acetone and embedded in Epon resin. Ultrathin sections obtained in an ultramicrotome were stained with uranyl acetate and lead citrate and examined in a Zeiss 900 transmission electron microscope.

### 3.11. Flow Cytometry

The *L. amazonensis* axenic amastigotes (5 × 10^6^ parasites/mL) were treated with **1** (50, and 100 μg/mL for 3 h at 32 °C), or untreated were harvested and washed with PBS. To gain analyses of mitochondrial membrane potential, (ΔΨm) parasites were stained with Rhodamine 123 (Rh 123) (5 mg/mL for 30 min at 37 °C) reagent following the protocol of manufacturer. The mean of fluorescence intensity of the cells was analyzed by flow cytometry FACSCalibur and CellQuest software). A total of 10,000 events were acquired in the region previously established as that corresponding to the parasites.

### 3.12. Measurement of Lipid Peroxidation Product

Samples of axenic amastigotes in exponential phase, were treated with 100 μg/mL of compound **1** for 6 h, at 32 °C. The thenoyltrifluoroacetone (TTFA) was used as positive control. After treatment, cells were washed with phosphate buffer, homogenized and added to a solution of 0.37% thiobarbituric acid in 15% trichloroacetic acid and 0.25 N HCl. The mixture was heated at 90–95 °C for 45 min. After cooling, butanol (1:1) was added to the solution. The mixture was shaken and centrifuged at 2000× *g* during 5 min. The optical density of the organic layer was determined at 535 nm in BIO-TEK Power Wave XS spectrophotometer. Lipid peroxidation was determined by the generation of thiobarbituric acid-reactive substances (TBARS) in terms of malondialdehyde (MDA), expressed in MDA nmol × protein mg^−1^ [41].

### 3.13. Statistical Analysis

All experiments were performed in duplicate. The means and standard deviations were determined from at least three experiments. Statistical analysis was performed with the program GraphPad Prism 4 (GraphPad Software, San Diego, California, USA). The student’s *t* test was applied, and a *p* value less than 0.05 were regarded as significant.

## 4. Conclusions

New antileishmanials from natural products are urgently needed due to the emergence of drug resistance in patients. In this context, the use of the compound **1** isolated from *C. cervicornis* against *L. amazonensis* parasites is of great interest. The *in vitro* treatment of the parasites with compound **1** showed notable ultra structural changes, displayed depolarization in the mitochondrial membrane potential, and an increase of lipid peroxidation. Although the mechanism of action of the compound **1** is still unclear, these findings appear to be a future alternative to development of new antileishmanial drugs.

## Figures and Tables

**Table 1:** Comparison between values of CC_50_ for J774G8 macrophages and IC_50_ for promastigote forms of *Leishmania amazonensis*, and their respective selectivity indexes (SI).

Compound	CC_50_ (μg/mL) a	Promastigote forms	

		IC_50_ (μg/mL)	SI
EACE	50.0 ± 0.08	50.0 ± 2.12	1.00
MCE	51.0 ± 0.20	100.0 ± 4.50	0.51
DCE	46.0 ± 0.46	20.0 ± 1.55	2.30
EAF	47.0 ± 0.75	8.0 ± 0.55	5.88
**1**	186.0 ± 3.29	2.0 ± 0.37	93.00
Amphotericin-B	nd	0.06 ± 0.00	nd

Values represent the mean ± S.D. of at least three experiments performed in triplicate.

a On macrophage strain J774G8 at 48 h of culture. SI = CC_50_ J774G8/IC_50_. nd: not determined.
